# Limberg flap reconstruction for recurrent pilonidal sinus disease: A single-center U.S. Cohort

**DOI:** 10.1007/s00423-026-03988-6

**Published:** 2026-02-12

**Authors:** Mustafa Oruc, Joshua Sommovilla, Salih Karahan, Joseph Trunzo, Metincan Erkaya, Michael Valente, Emre Gorgun

**Affiliations:** Digestive Disease Institute, Department of Colorectal Surgery, Cleveland Clinic, Cleveland, OH USA

**Keywords:** Recurrent pilonidal disease, Limberg flap surgery, Postoperative outcomes

## Abstract

**Purpose:**

Recurrent pilonidal sinus disease (PSD) presents a significant surgical challenge. Although Limberg flap (LF) reconstruction has demonstrated favorable outcomes in the international literature, long-term outcome data from U.S. institutions, particularly in recurrent cases, remain limited. This study evaluated the long-term outcomes of LF reconstruction in a single-center U.S. cohort with recurrent PSD.

**Methods:**

A retrospective analysis was conducted of patients who underwent LF for recurrent PSD between 2014 and 2023. Data on patient demographics, operative details, and postoperative courses were collected. Long-term follow-up, including assessment for recurrence, was obtained via structured telephone interviews and chart reviews.

**Results:**

Thirty-seven patients were included. The cohort was predominantly male (76%) with a median BMI of 29.4 kg/m². 59% were active smokers, and 52% had undergone multiple previous pilonidal operations. The median operative time was 72 min. 92% of the patients were discharged on the same day, and no surgical drains were used. The postoperative complication rate was 11%, primarily consisting of wound dehiscence. No recurrence was observed during the median follow-up of 4.5 years.

**Conclusion:**

In this retrospective cohort study of patients with recurrent pilonidal sinus disease, Limberg flap reconstruction was associated with durable disease control and acceptable postoperative morbidity while remaining compatible with early discharge. These findings support Limberg flap reconstruction as a viable option for patients with complex recurrent disease.

## Introduction

Pilonidal sinus disease (PSD) is a chronic, debilitating condition that commonly affects young adults, particularly males [1]. It is characterized by the presence of a sinus tract or cyst in the sacrococcygeal region and is frequently associated with recurrent infections, abscess formation, and persistent discomfort. These features can significantly reduce the quality of life of patients and contribute to substantial morbidity [2]. Despite the availability of various treatment options, recurrence remains a major challenge, particularly in patients who have undergone multiple surgical interventions [3].

Several surgical techniques have been employed to manage PSD, including wide excision with secondary healing, primary midline closure, and various flap-based reconstructions [4]. Among these, the Limberg flap (LF), first described by Alexander Limberg in 1948, has gained popularity owing to its geometric design, which facilitates tension-free closure and lateralization of surgical wounds. Numerous studies, particularly those from Europe and the Middle East, have reported favorable outcomes of LF reconstruction [5]. However, these studies often included mixed cohorts of primary and recurrent diseases, and only a limited number focused exclusively on recurrent cases [6, 7].

Additionally, the current evidence base is geographically narrow, with virtually all data originating from non-North American populations. The few available North American studies have mainly evaluated primary cases or compared other surgical methods [8], without a dedicated analysis of surgically complex, previously operated patients [9, 10]. Given the differences in surgical practice patterns, perioperative care, and patient demographics across regions, a significant and clinically relevant gap exists in the literature [11].

This gap in regional evidence is particularly important, given the unique challenges posed by recurrent cases of pilonidal disease. These cases involve altered anatomy, extensive scarring, and an elevated risk of wound-related complications, often necessitating technically demanding surgical management. Therefore, this study aimed to evaluate the surgical and long-term outcomes of LF reconstruction in a North American cohort of patients with recurrent pilonidal sinus disease.

## Materials and methods

### Patient selection

This cohort included all consecutive patients with recurrent pilonidal sinus disease who underwent LF reconstruction between 2014 and 2023. During the study period, off-midline flap reconstruction was routinely used for surgical management of recurrent pilonidal sinus disease at our institution. LF reconstruction was the most commonly performed technique for recurrent disease, with procedure selection guided by patient characteristics and surgeon judgment. The procedures were performed by two attending surgeons (EG and JS) in a single colorectal surgery practice. Patients who underwent primary surgery, those treated with alternative surgical techniques, and those with insufficient follow-up were excluded.

### Outcome definitions

The primary outcome was disease recurrence. Recurrence was defined as the return of pilonidal disease, including the development of new sinus tracts, cysts, or abscesses, at a surgical site that had previously achieved complete wound healing and required additional medical or surgical treatment. Wound complications, including infection or wound dehiscence occurring prior to complete or durable wound healing, were not classified as recurrence.

Complete wound healing was defined as full epithelialization of the surgical site following suture removal with no requirement for additional wound care or dressings. Postoperative complications were defined as any deviation from the expected postoperative course within 30 days of surgery, including wound dehiscence, hematoma, seroma, or delayed wound healing. Healing time was defined as the interval between surgery and the first documented confirmation of complete wound healing. The number of postoperative office visits was defined as the total number of in-person outpatient evaluations performed by the surgical team from surgery until complete wound healing, including wound assessment, dressing changes, complication monitoring, and suture removals.

### Data collection

Relevant data were extracted from the electronic medical records. Collected variables included patient demographics, prior surgical history (type, number, and timing of previous procedures), operative details (operation time and intraoperative findings), and postoperative outcomes (length of hospital stay, complications, number of office visits, healing time, and recurrence). Recurrence was assessed by reviewing medical records and conducting telephone interviews.

### Surgical technique

All procedures were performed under general anesthesia, with the patient in the prone jackknife position. Intravenous prophylactic antibiotics were administered preoperatively according to the institutional surgical site infection prevention protocols. The gluteal region was shaved, prepped, and draped in a sterile manner.

**Fig. 1:**
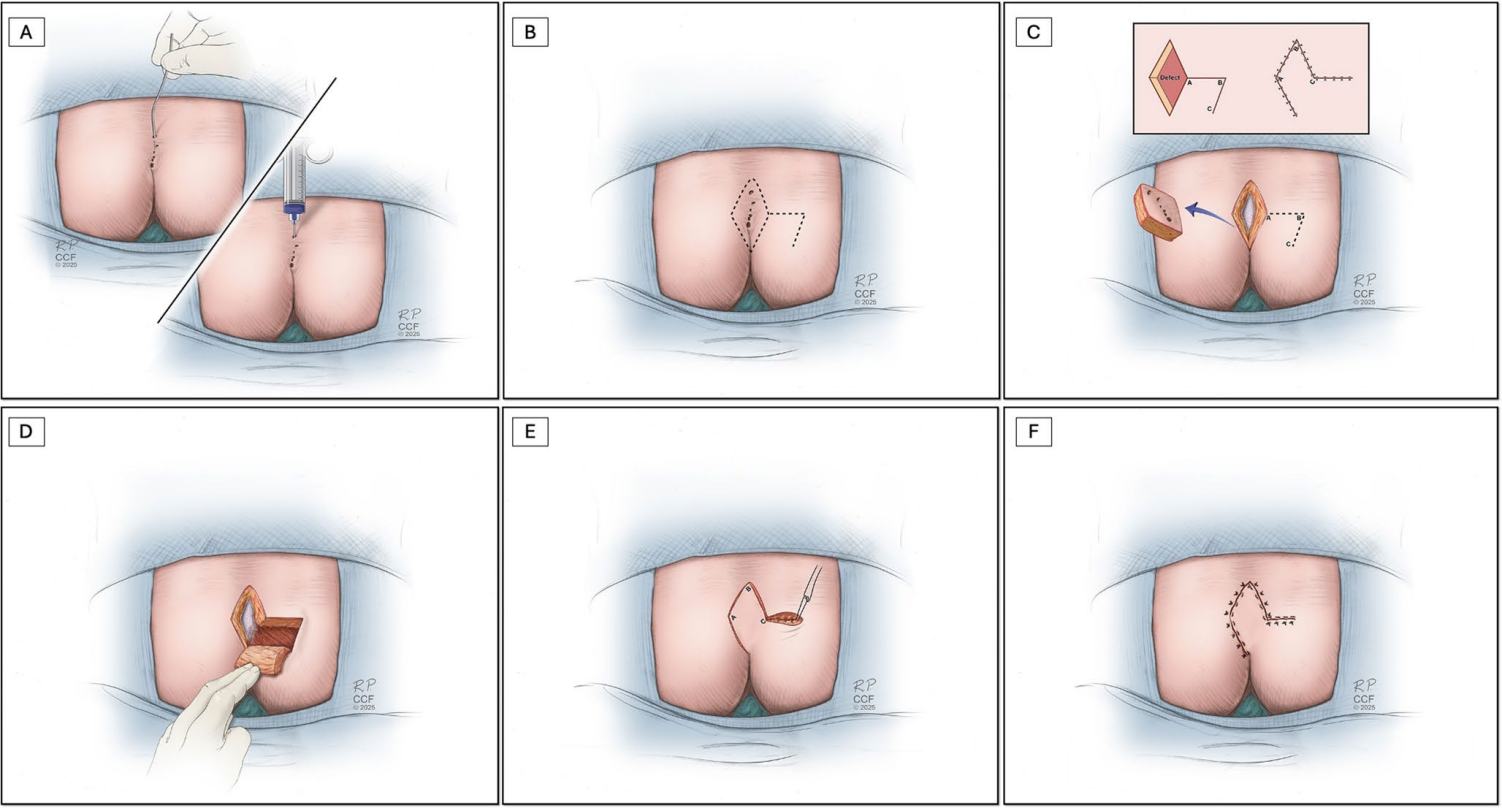
Surgical steps of Limberg flap reconstruction for pilonidal disease. (**A**) Stylet use and methylene blue injection to assess sinus depth; (**B**) marking of rhomboid flap lines; (**C**) excision of diseased tissue; (**D**) flap creation; (**E**) medialization and suturing of the flap; (**F**) final appearance after reconstruction

Sinus pits were carefully identified with a stylet, and 1:1 saline-diluted methylene blue dye was injected through a 14 or 16 gauge angiocath to delineate the full extent of the sinus tract and any lateral extensions. A rhomboid-shaped area encompassing the entire diseased tissue was marked for excision. The rhomboid design featured medial and lateral angles of approximately 120° and superior and inferior angles of 60°, which facilitated flap creation and tension-free closure (Fig. 1A–B).

Complete excision of all involved skin, sinus tracts, subcutaneous tissue, and pre-sacral fascia was performed, ensuring complete removal of the infected and fibrotic tissue. Hemostasis was achieved using electrocautery, and the surgical field was irrigated with sterile saline (Fig. 1C).

A Limberg transposition flap of equal dimensions was designed adjacent to the rhomboid defect. The flap was drawn as a continuation of the rhomboid excision, extending laterally from the inferolateral border of the defect (Fig. 1C). The horizontal line should be at least 60% of the vertical defect height. The angles and dimensions were replicated to ensure symmetry and allow tension-free transposition into the primary defect.

The flap was elevated in the subfascial plane, preserving the integrity of the subdermal vascular plexus and surrounding tissue. Dissection proceeded from lateral to medial, and care was taken to maintain a generous pedicle and avoid kinking or torsion during flap mobilization. The flap consisted of the epidermis, dermis, subcutaneous tissues, and gluteal muscle fascia (Fig. 1D). Once fully elevated, the flap was rotated medially and transposed into the primary defect to provide full coverage of the presacral space (Fig. 1E).

The wound was closed in three layers. The deep fascia of the flap was anchored to the sacral fascia using interrupted 2 − 0 Vicryl sutures (Ethicon, Somerville, NJ, USA). The subcutaneous tissue was approximated using 3 − 0 and 4 − 0 Vicryl sutures in a layered manner. The skin was closed using 3 − 0 interrupted Prolene sutures (Ethicon, Somerville, NJ, USA) to ensure secure and tension-free approximation of the wound edges (Fig. 1E-F).

### Postoperative care and follow-up

Patients were discharged on the same postoperative day or on day one. No drain was used. Patients were instructed to avoid sitting or lying directly on the flap for at least two weeks and to limit physical activity for six to eight weeks to reduce the risk of flap failure and wound complications. Adequate pain control was provided according to the institutional protocol. Antibiotics are not routinely prescribed unless there are concerns regarding postoperative infection or cellulitis.

During the first postoperative week, all patients underwent in-office evaluation for early complications, including seroma formation or surgical site infection. The sutures were removed at the second follow-up visit, typically between postoperative day 14 and 21. Additional complications were assessed and managed on a case-by-case basis.

### Statistical analysis

Descriptive statistics were used to summarize the patient characteristics and clinical outcomes. Continuous variables were reported as medians with interquartile ranges (IQR), depending on the data distribution. Categorical variables were presented as counts and percentages. All statistical analyses were performed using R software (version 4.4.1).

### Ethical consideration

This study adhered to the ethical principles of the Declaration of Helsinki. Ethical approval was obtained from the Institutional Review Board (IRB) of the Cleveland Clinic.

## Results

During the study period, 43 patients with recurrent pilonidal sinus disease underwent LF reconstruction at our institution. Of these, six patients were excluded due to insufficient follow-up data. Therefore, the final study cohort comprised 37 patients. Of these, 28 (76%) were men and nine (24.3%) were women. 34 (92%) patients were identified as White, and three (8%) were identified as Black. The median age of the cohort was 26 years (interquartile range [IQR], 21–30 years), and the median body mass index (BMI) was 29.6 (IQR, 25.1–34.2) kg/m². Twenty-two patients (59%) were active smokers, and two patients (5%) had a history of diabetes mellitus. None of the patients had a history of steroid use (Table 1).

**Table 1:** Summary of limberg flap surgery outcomes for recurrent pilonidal disease

Category	Value (*n* = 37)
Demographics	
Gender (male), n,(%)	28 (75.6)
Race, n (%) White Black	34 (92) 3 (8)
Age, years, median (IQR)	25 (21–30)
BMI, kg/m², median (IQR)	29.6 (25.1–34.2)
Active smokers, n (%)	22 (59)
Diabetes mellitus, n (%)	2 (5)
Clinical History Previous operation type, n (%)	
Previous excision with closure	24 (65)
Previous excision with marsupialization	13 (35)
Interval since last surgery, years, median (IQR)	0.94 (0.41–2.98)
Recurrence timing n, (%) <12 months 12–24 months 24 < months	20 (54) 10 (27) 7(19)
Recent abscess drainage (< 6 months), n (%)	12 (33)
≥ 2 prior operations, n (%)	19 (52)
Surgical Details	
Intraoperative localized abscess, n (%)	7 (19)
Operative time, min, median (IQR)	72 (49–79)
Same day discharge, n (%)	34 (92)
Postoperative Outcomes	
Post-op office visit count, n, median (IQR)	2 (1–3)
Wound healing time, days, median (IQR) Without wound dehiscence With wound dehiscence	26 (19–42) 150 (96–277)
Post-op complications (wound dehiscence), n (%)	4 (11)
Long-term Outcomes	
Follow-up duration, years, median (IQR)	4.5 (3.3–6.9)
Recurrence, n (%)	0 (0)

BMI: Body Mass Index; IQR: Interquartile range

Regarding surgical history, 24 patients (65%) had previously undergone primary excision and midline closure, whereas the remaining 13 (35%) had undergone primary excision with marsupialization or secondary healing. The median interval since the last surgical intervention for pilonidal disease was 0.94 years (IQR, 0.41–2.98 years). Recurrence following previous procedures occurred within the first year in 20 patients (54%), between 12 and 24 months in ten patients (27%), and after 24 months in seven patients (19%). Twelve patients (33%) had undergone abscess drainage other than their primary surgery within the six months preceding surgery, and 19 patients (52%) had a history of more than one prior operation. Intraoperatively, seven patients (19%) were found to have localized deep abscesses in the sinus cavity.

The median operative time for the LF procedure was 72 min (IQR, 49–79 min). 92% of the patients (*n* = 33) were discharged on the same day. Postoperative recovery was generally favorable, and patients had a median of two postoperative office visits (IQR, 1–3 times). The median time to complete wound healing was 26 days (IQR, 19–42 days), which was prolonged to 150 days (IQR, 96–277 days) in patients who developed wound dehiscence prior to complete healing.

Four patients (11%) experienced postoperative complications, all of which involved wound dehiscence. One patient developed a hematoma in the early postoperative period, which was managed with local drainage and subsequently treated with vacuum-assisted closure (VAC) therapy and local wound care. The second patient developed a 3.5 cm wound dehiscence that was managed with regular dressing and packing, achieving secondary closure without the need for VAC therapy. The third patient presented with late-onset dehiscence three months after surgery and required surgical debridement, followed by VAC therapy. The fourth patient presented with chronic infection at the time of surgery and was found to have a localized deep abscess within the sinus cavity intraoperatively. In the early postoperative period, the patient developed a 2 cm wound dehiscence, which was managed conservatively with routine dressing changes.

The median follow-up period was 4.5 years (IQR, 3.3–6.9), and no recurrence was observed during the follow-up period.

## Discussion

This study evaluated the outcomes of LF reconstruction in a challenging, high-risk cohort of patients with recurrent pilonidal sinus disease, characterized by complex disease and multiple prior surgical interventions, at a single referral institution. All patients achieved complete wound healing, and no disease recurrence was observed during the median follow-up of 4.5 years. Postoperative morbidity was limited to wound-related complications, which were successfully managed and did not progress to recurrent disease. Although data on LF reconstruction are available in the international literature, long-term outcome data from U.S. institutions remain scarce; therefore, these findings add institution-level evidence supporting the durability of this technique in a difficult-to-treat population.

Recurrence remains a central outcome in the evaluation of pilonidal sinus disease; however, reported recurrence rates vary widely across the literature [12]. This variability reflects substantial heterogeneity in patient populations, surgical techniques, follow-up duration, and definitions used to classify recurrence [13]. Many studies combine primary and recurrent disease, apply variable temporal thresholds, or fail to clearly distinguish disease recurrence from early surgical failure or delayed wound healing [11, 14]. Recurrence risk has been associated in prior studies with patient- and disease-related characteristics, including smoking, obesity, family history of pilonidal disease, and prior surgical intervention; however, the prevalence and strength of these associations vary across reported cohorts [10, 15, 16]. To allow for a clear differentiation between true disease recurrence and postoperative wound-related complications, the present study defined recurrence as the reappearance of pilonidal disease at a site that had achieved complete healing following definitive intervention. Time-based definitions of recurrence, such as those requiring a fixed postoperative interval (e.g., 30 days), may be appropriate for limited excision strategies but are less applicable to wide excision or lay-open techniques, where prolonged healing times are common [10, 17–19].

Prior surgical intervention has been shown to confer a higher risk of recurrence and distinct recurrence patterns in pilonidal sinus disease, underscoring the intrinsic complexity of recurrent cases and the need for durable surgical strategies [20]. Off-midline flap-based reconstructions are considered advanced surgical approaches and, according to current guidelines, are primarily indicated for complex or recurrent pilonidal sinus disease [12, 21, 22]. Among these techniques, the LF is one of the most clearly defined and extensively evaluated [23]. Randomized trials and meta-analyses have shown that LF reconstruction is associated with lower wound-related complication rates than midline closure. Recurrence rates are generally comparable to those of other off-midline closure techniques [5, 24]. Modifications to the classic Limberg design, aimed at further lateralizing the incision and suture line away from the midline, have been developed to optimize outcomes, and meta-analyses suggest that these modified approaches may achieve the most favorable profiles with respect to both recurrence and postoperative morbidity [25, 26]. Despite this robust body of evidence, the adoption of LF reconstruction in North American surgical practice remains limited, with only one small published series reporting no recurrence but frequent wound-related complications [27]. Therefore, the present study contributes longitudinal outcome data from a United States referral population, supporting the durability and clinical utility of the LF in the management of complex, recurrent disease.

The technical demands of off-midline flap reconstruction, coupled with the inherent complexity of recurrent disease, account for a documented spectrum of postoperative complications following LF surgery, ranging from minor seromas to wound infection and wound dehiscence. Reported rates of such wound-related complications vary substantially across published series, with dehiscence cited between two and forty-five% [28, 29]. The majority of these events are minor and manageable with conservative measures, whereas serious adverse events, such as flap necrosis or the need for reoperation, remain rare, even in high-risk populations [30, 31]. In the present series, the postoperative complication rate was 11%. Although these events were associated with prolonged wound healing, none represented severe morbidity or progressed to disease recurrence. Historically, this procedure has been associated with extended hospitalization and routine drainage placement [23, 32, 33]. In contrast, in this cohort, most patients were discharged on the same day without surgical drains, with no apparent increase in clinically significant complications or recurrence. These findings suggest that LF reconstruction can be performed with manageable morbidity while remaining compatible with early discharge.

This study is strengthened by the inclusion of a well-defined cohort of patients with complex recurrent pilonidal sinus disease and long-term follow-up and by providing additional data on an underutilized surgical technique in the United States from a tertiary referral setting. Several limitations must also be acknowledged, including the retrospective design, moderate sample size, single-center setting, and absence of a comparative surgical group, all of which constrain direct comparisons or causal inferences. Future multicenter studies should incorporate standardized definitions of recurrence and disease complexity, distinguish primary from recurrent disease, and focus on outcomes in challenging, referral-based populations to better define optimal surgical strategies for pilonidal sinus disease.

## Conclusion

In this retrospective cohort study of patients with recurrent pilonidal sinus disease, Limberg flap reconstruction was associated with durable disease control and acceptable postoperative morbidity while remaining compatible with early discharge. These findings add long-term outcome data from a U.S. referral cohort and support Limberg flap reconstruction as a viable option for the management of complex recurrent disease.

## Data Availability

The datasets generated during and/or analyzed during the current study are not publicly available due to patient privacy and ethical restrictions but are available from the corresponding author upon reasonable request. Data sharing will be subject to a formal data use agreement and approval from the Cleveland Clinic Institutional Review Board.
